# Diarrheal disease and associated factors among children aged 6 to 59 months in Oda Bultum District, Eastern Ethiopia: a community-based cross-sectional study

**DOI:** 10.1186/s12879-024-09169-4

**Published:** 2024-03-12

**Authors:** Zewudalem Getachew, Nega Asefa, Tigist Gashaw, Abdi Birhanu, Adera Debella, Bikila Balis, Usmael Jibro, Sina Tolera, Aboma Motuma, Mulugeta Gamachu, Alemayehu Deressa, Fethia Mohammed, Moti Tolera, Addis Eyeberu, Lemma Demissie Regassa, Ibsa Mussa

**Affiliations:** 1 Oda Bultum Woreda Health Office, Oda Bultum, West Hararghe, Oromia, Ethiopia; 2 School of Public Health, College of Health and Medical Sciences, Haramaya University, Harar, Ethiopia; 3 School of Nursing and Midwifery, College of Health and Medical Sciences, Haramaya University, Harar, Ethiopia; 4 School of Medicine, College of Health and Medical Sciences, Haramaya University, Harar, Ethiopia; 5 Department of Psychiatry, School of Nursing and Midwifery, College of Health and Medical Sciences, Haramaya University, Harar, Ethiopia; 6 Department of Environmental Health Sciences, College of Health and Medical Sciences, Haramaya University, Harar, Ethiopia; 7 School of Pharmacy, College of Health and Medical Sciences, Haramaya University, Harar, Ethiopia

**Keywords:** Associated factors, Diarrhea, Eastern Ethiopia, Under five children, Vaccination

## Abstract

**Background:**

Diarrhea is a serious health problem in children under the age of five that is both preventable and treatable. In low-income countries like Ethiopia, children under the age five years frequently experience diarrhea. However, the burden and associated factors of these diarrheal diseases are understudied in Eastern Ethiopia, Thus, this study aimed to determine the factors associated with the prevalence of diarrheal diseases in Eastern Ethiopia from September 1–30, 2022.

**Methods:**

A cross-sectional study was conducted on the total of 602 children aged 6 to 59 months in Oda Bultum district in eastern Ethiopia. A multistage sampling method was used. Three kebeles were selected from nine kebeles by the lottery method. Data was entered into Epi data 4.0.2 and exported to SPSS version 21 for analysis. Descriptive analysis was used for frequency, mean, and standard deviations. In addition, bivariable, and multivariable Poisson regression model was used to identify predictors of diarrhea along with a 95% confidence interval. Finally, statistical significance was declared at a p-value of 0.05.

**Result:**

A total of 602 children were included in this study. The prevalence of diarrhea 7.4% (47/602), 95% CI; 5.5–9.7%) among the children. Factors such as being unvaccinated for any vaccine (AOR = 10.82, 95%CI; 4.58–25.48) and born from a mother who had medium level of empowerment (AOR = 0.34, 95%CI; 0.11–0.88) in the household had statistically significant association with diarrhea among the children compared to their counterparts.

**Conclusion:**

The study found that nearly one out of thirteen children aged 6 to 59 months had any form of diarrheal diseases in Oda Bultum District, Eastern Ethiopia. In addition, the study revealed that children who were vaccinated for their age developed diarrhea less likely compared to those who did not receive any form of vaccine for their age. Moreover, children with mothers who had a medium level of empowerment were less likely to get diarrhea than children with mothers who had a low level of empowerment.

## Introduction

Diarrheal disease is a serious health problem that is both preventable and treatable but It can cause dehydration, malnutrition, and death among all age if not managed well [1–3]. World Health Organization (WHO), reported there are an estimated 1.7 billion of diarrheal disease cases each year, resulting in half a million deaths [3]. Most of these deaths occur in children under the age of five in developing countries [3]. Diarrheal disease is the second leading cause of death in children under five years old [1, 4]. In developed countries, the incidence of diarrheal disease is lower, but it still remains a significant health concern [5].

Children under the age of five in low-income countries often get three bouts of diarrhea each year where each episode of diarrhea deprived the child of the nourishment necessary for growth [6]. As a result, diarrhea contributed to undernutrition, and made them more susceptible to diarrheal diseases [2]. Diarrheal disease, which is characterized by loose, watery stools is a common ailment that affects people of all ages and backgrounds, can be caused by a variety of factors, including viral, bacterial, and parasitic infections, and most commonly transmitted by contaminated food or water [2, 3]. Globally, 780 million individuals lack access to improved drinking water and 2.5 billion lack improved sanitation as such diarrhea due to infection is widespread throughout developing countries [3].

Evidence showed the magnitude of diarrheal disease in Ethiopia varied. Systematic reviews and meta-analyses of studies conducted in Ethiopia reported that the pooled prevalence of diarrheal disease in Ethiopia ranges from 19 to 25% [7]. A global burden of disease reported that the prevalence of diarrheal disease in under-5 (13.2%) and is the second cause of death [8]. Another study from Sidama region; southern Ethiopia reported the prevalence of diarrheal disease among under-five children ranged between 10.7 and 16.5% [9]. A study conducted in Jimma also reported sociodemographic, environmental, immunization status and behavioral factors are the main risk factors for diarrheal disease [10]. The same study also reported that children who were not vaccinated against rotavirus were about 2.5 times at risk of having diarrheal disease [10].

A significant proportion of diarrheal disease can be prevented through safe drinking water, adequate sanitation and hygiene and vaccination. Vaccination is one of the most effective ways to reduce child mortality and morbidity from diarrheal disease, which is caused by various infectious agents like rotavirus, cholera, typhoid, and other enteric pathogens can protect children from severe and life-threatening diarrhea [1, 11]. These vaccines are the most effective and a key component of primary health care and an indisputable human right [12]. A study conducted in Ethiopia also supported the above evidences as the introduction of the vaccine led to a significant reduction in hospitalizations due to rotavirus diarrhea [13].

Global reports showed that significant progress has been made in reducing child mortality globally Between 1990 and 2017. Neonatal Mortality Rate decreased by 51%, from 36·6 deaths per 1000 live births in 1990, to 18·0 deaths per 1000 live births in 2017 [14]. However, focused efforts are still needed in sub-Saharan Africa to prevent deaths, of which diarrheal disease is the leading cause [15]. Hence, it is essential to monitor the risk factors for diarrheal disease to develop and test new diarrhea prevention and control strategies [16]. Despite the higher prevalence, there were fewer studies conducted in eastern Ethiopia especially in Eastern Oromia. Therefore, to support this effort, this study aimed to determine the factors associated with the diarrheal diseases among children in eastern Ethiopia.

## Methods and materials

### Study setting, period and design

A cross-sectional study among women having children between 6 and 59 months of age and their children was conducted in selected kebeles (the smallest administrative division of Ethiopia) of Oda Bultum district in West Hararghe zone Ethiopia from September 1–30, 2022. Oda Bultum district has 9 kebeles and is located 331 km east of Addis Ababa and 37 km from Chiro town, the zonal town of the Western Hararghe zone. The district has 39 health posts, 6 health centers, and 6 private clinics.

### Population and eligibility criteria

Mothers having children aged between 6 and 59 months were included. The selected children’s mothers were interviewed. Children with deformities and birth defects were excluded to avoid comparison bias as the deformities hamper the measurements.

### Sample size and sampling procedure

The sample size was calculated using the single population proportion formula with the assumptions of prevalence of diarrhea among children(*P* = 24%) [17], q = 1-p(1-0.24) = 0.76, and 5% of margin of error(d) at 95% confidence interval(z = 1.96). Finally, by considering the multistage sampling design, a design effect for the handling of intraclass variation was determined to be 2. The formula used for sample size calculation was as follows:

$${\text{n}}\,{\text{=}}\,{\left( {{\text{Z@}}\,{\text{/}}\,{\text{2}}} \right)^{\text{2}}}\,\left( {{{\text{p}}^ * }{\text{q}}} \right)\,{\text{/}}\,{{\text{d}}^{\text{2}}}$$

$${\rm{n}}{\mkern 1mu} {\rm{ = }}{\mkern 1mu} {\left( {{\rm{1}}{\rm{.96}}} \right)^{\rm{2}}}{\mkern 1mu} \left( {{\rm{0}}{\rm{.24}}} \right){\mkern 1mu} \left( {{\rm{0}}{\rm{.76}}} \right){\mkern 1mu} {\rm{/}}{\mkern 1mu} {\left( {{\rm{0}}{\rm{.05}}} \right)^{\rm{2}}}$$

**Fig. 1:**
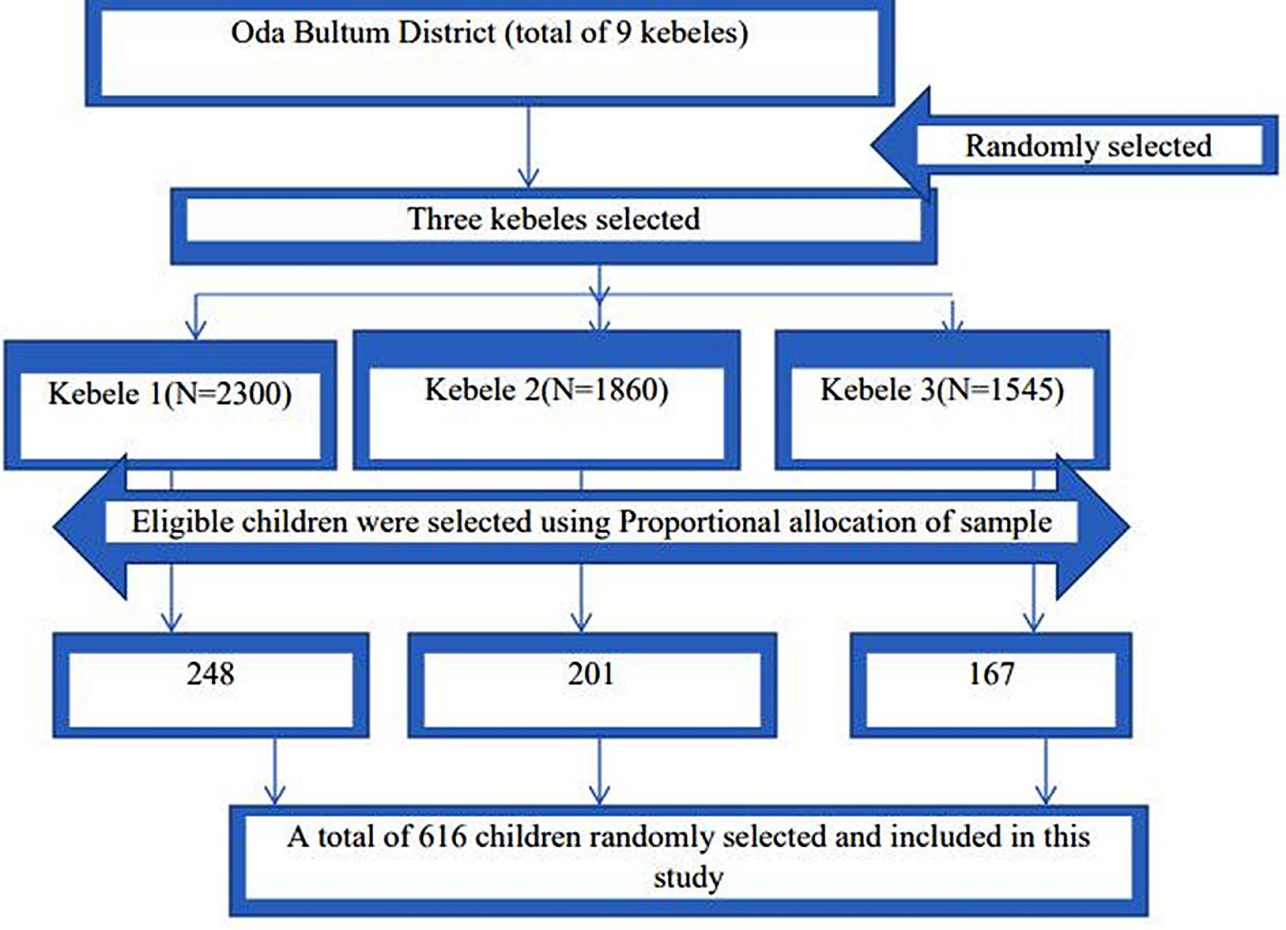
Sampling procedure of study participants’ selection among under-five children in Oda Bultum District, 2022

*n* = 280, by considering the design effect (DE) 2, the sample size was determined to be 560. Finally, by considering 10% of non-response rate, the sample size became 616. Regarding the sampling procedure, three of 9 kebeles in Oda Bultum district were randomly selected. To enroll all samples, the proportional allocation of sample was applied to all the three selected kebeles based on the total number of eligible children. According to the demography of the district, there were a total of 32,857 children aged 6–59 months in the district. Using their list of the health posts, the total samples were selected using simple random sampling by lottery method (Fig. 1).

### Data collection methods and tool, and data quality control

A structured questionnaire was adapted from different literature, including sociodemographic variables, child nutritional status, variables like wasting, stunting, and undernutrition, women’s empowerment variables, and maternal-related factors such maternal age, marital, and education. Then the questionnaire was translated into Afaan Oromo by a person who speaks both languages and has knowledge of the subject matter. The questionnaires were pretested on 5% of the total sample size in the nearby district called Gemechis. A face-to-face interview with a structured questionnaire and anthropometric measurements was done by trained data collectors to collect data. Eight data collectors who can speak the Afan Oromo language and two supervisors with a BSc in public health officers or nurses were recruited for data collection. Daily supervision and follow-up were done by the supervisors and principal investigators. The vaccination history of the children was validated by checking against immunization cards.

### Study variables and measurements

Diarrheal disease was an outcome variable in this study. Independent variables included level of women’s empowerment, socio-demographic variables (age, religion, educational status, occupational status, marital status, husband’s educational status, and husband’s occupation), food production and prices, household and non-household food expenditure, access to health care and information, caring capacity of the women, diet, health status of the child and the mother, and household decision-making.

### Operational definitions

#### Women empowerment

A process through which women and girls acquire knowledge, skills, and willingness to critically analyze their situation and take appropriate action to change the status quo of women and other marginalized groups in society [18].

#### Vaccinated for age

We categorize the child as vaccinated to the age when the child has received all recommended vaccines for their age group. Vaccines are important to protect children from serious and sometimes dead diseases, such as measles, polio, tetanus, and meningitis, considering different vaccines are given at different ages, depending on the child’s risk of exposure and immune system development [19].

#### Timely vaccination

was measured if a child was vaccinated within one month after the minimum age to administer the dose as recommended by WHO [20–22]. In addition, in this study, the “vaccinated for age” term is used to assess whether a child took a vaccine recommended for his/her age.

#### Diarrhea

was defined as passage of at least three loose or watery stools per day among the children in the household before the data collection, as reported by the caregiver or mother of the child [23, 24].

### Data processing and analysis

Data were entered into Epidata version 4.0.2 and exported to SPSS version 21 for analysis. Descriptive analysis like percentage, means, and standard deviation were calculated as necessary. Child nutritional status was measured by using anthropometric measurements for wasting weight, for height, for underweight (WAZ), and for stunting (HAZ). Children wt-for-age Z-Score (WAZ) was below minus two standard deviations (-2 SD) from the median of the WHO reference population were classified as underweight. Children whose height-for-age Z-score (HAZ) was below − 2 SD from the median of the WHO reference population were considered short for their age (stunted) or chronically malnourished [25–27].

The level of women’s empowerment was measured by women’s involvement in household decision-making (including 3 decisions: access to health care, household purchasing, and freedom to visit relatives, rated as follows: participated in all 3 decisions = 2, participated in 1 or 2 decisions = 1, did not participate in any decisions = 0); women’s membership in community groups (member of any community group rated by); Was not involved in any groups = 0); women’s cash earnings measured by (earning cash only or both cash and in-kind =, did not earn cash at all = 0); women’s ownership of house or land (owned a house or land, alone or jointly with husband = 1, did not own any house or land = 0); women’s education (attained secondary or higher education = 2, attained primary level education = 1, did not attend school at all = 0); And finally, the total score was summed from 0 to 7, and the scores from 0 to 2 are grouped as low empowerment level; the scores from 3 to 4 are grouped as moderately empowered level; and the scores from 5 to 7 are grouped as highly empowered level [28].

In the Bivariable analysis, the variables that had P-value less than 0.3 were transferred to multivariable analysis model. Multicollinearity was diagnosed using VIF and tolerance. Finally, the final model, multivariable Poisson regression was used to identify predictors of diarrheal disease among children and. Finally, statistical significance was declared at a p-value of 0.05 at 95% confidence interval.

## Results

### Socio-demographic characteristics of the study participants

A total of 602 children with a response rate of 97.7% were included in this study. Three-fifths of the children’s families were Muslims, whereas the rest 224(36.1%) of them were Christians. A total of 584(94.2%) mothers were housewives, while the rest were gov’t workers, farmers, and merchants. More than half, 370(59.6%) of the children’s fathers were unable to read and write and used farming as their primary occupation. About 250(40.3%) of the households had more than family members. Nearly three out of five households used to drink from an improved drinking water source, a total of 407(65.6%) households used latrines regarding the accessibility of the health facilities, 386(62.3%) of the households accessed the health facility by walking for above 30 min (Table 1).

**Table 1:** Socio-demographic characteristics of study participants, Oda Bultum, Oromia, Ethiopia, 2022

Variables	Category	Frequency	Percent
Religion	Christian	224	36.1
	Muslim	396	63.9
Maternal occupation	Housewife	584	94.2
	Gov’t employee	15	2.4
	Farmer	10	1.6
	Merchant	11	1.8
Father education	Illiterate	370	59.6
	Primary	247	39.8
	Secondary	3	0.5
Father occupational status	Farmer	573	92.4
	Daily laborer	24	3.8
	Merchant	20	3.2
	Others	3	0.4
Family size	Less than five	146	23.5
	Five	224	36.1
	Above five	250	40.3
Source of drinking water	Improved	366	59
	Unimproved	254	41
Having latrine	No	34	5.5
	Yes, but used yet	179	34.4
	Yes	407	65.6
Accessibility of *HF	Within 30 min	234	37.7
	Above 30 min	386	62.3

### Child and maternal characteristics

More than half, 336(54.2%) of the children were females. About 267(43.1%) of the children were breastfeeding during the time of data collection. More than half, 334(53.8%) of the children initiated complementary feeding at the age of 6 months. About 565(91.1%) of the children were vaccinated for their age. Nearly three-fourths of the children’s mothers were in the age category of 25 to 34 years. Most of the maternal marital status was married. Nearly half of the mothers had low empowerment scores in their households (Table 2).

**Table 2:** Maternal and child characteristics of study participants, Oda Bultum, Oromia, Ethiopia, 2022

Variable	Category	Frequency	Percent
Child sex	Male	284	45.8
	Female	336	54.2
Breastfeeding	No	353	56.9
	Yes	267	43.1
When initiated complementary feeding?	Before 6 months	95	15.3
	At 6 months	334	53.8
	After 6 months	191	30.8
Vaccinated	Yes	565	91.1
	No	55	8.9
Maternal age	15–24	81	13.1
	25–34	456	73.5
	35–44	83	13.4
Child age	6–23 months	327	52.7
	24–59 months	293	47.3
Maternal empowerment status	Low	306	49.3
	Medium	238	38.4
	High	76	12.3
Maternal Marital status	Married	599	96.6
	Widowed	9	1.4
	Divorced	12	1.9

### Distribution of diarrheal among under-five children

**Fig. 2:**
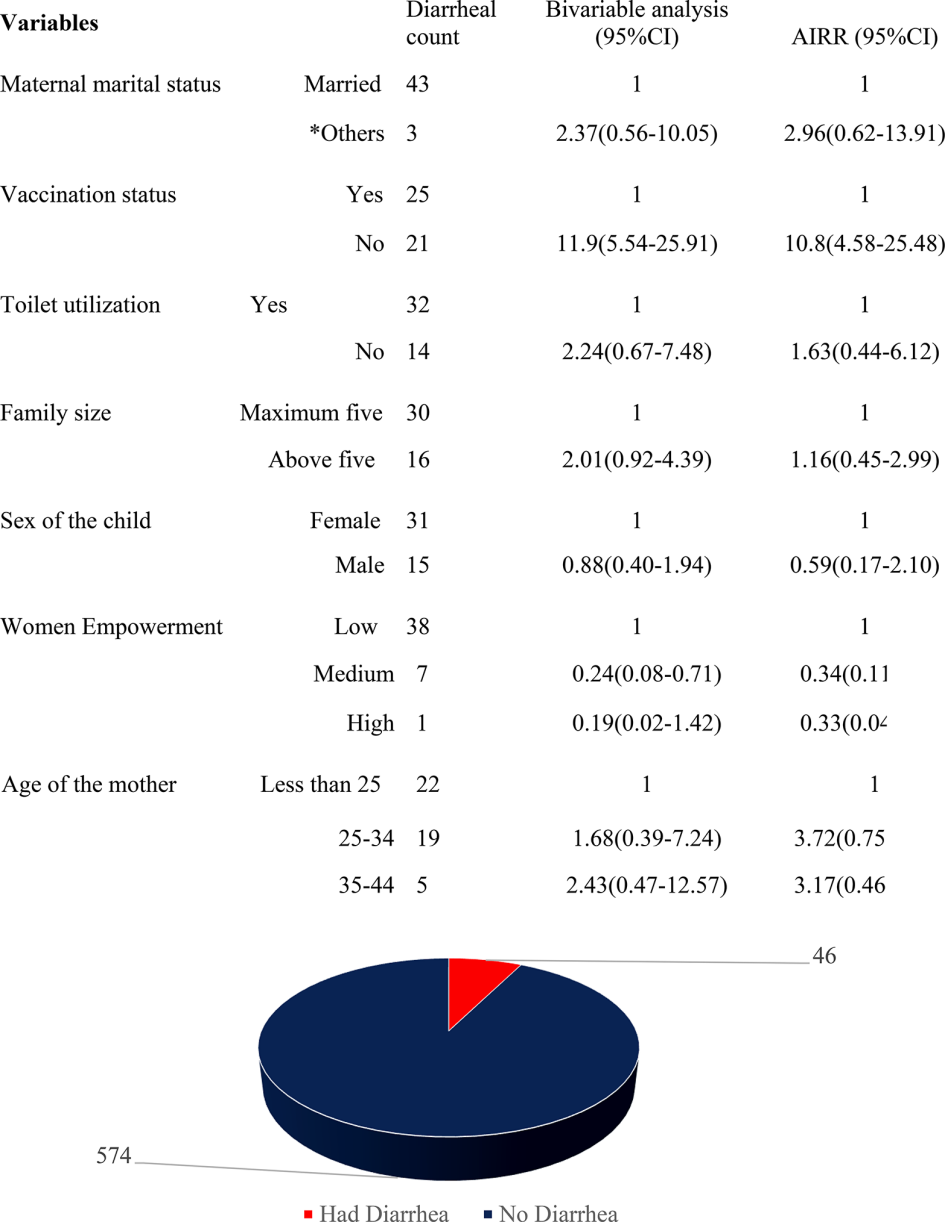
Magnitude of diarrhea among under-five diarrhea, Oda Bultum, Oromia, Eastern Ethiopia, 2022

Diarrhea episodes were detected among 46 (7.4%, 95% CI; 5.5–9.7%) among the children (Fig. 2).

### Factors associated with diarrheal disease

In bivariable analysis, the variables that had a P-value less than 0.3 were transferred to the multivariable Poisson regression model. Finally, the vaccination status and maternal empowerment status were statistically associated with diarrheal episodes among children (*P* < 0.05) (Table 3).

**Table 3:** Multivariate logistic regression analysis of factors associated with diarrheal disease, Oda Bultum, Oromia, Eastern Ethiopia, 2022

Variables		Diarrheal count	Bivariable analysis (95%CI)	AIRR (95%CI)	P-value
Maternal marital status	Married	43	1	1	
	*Others	3	2.37(0.56–10.05)	2.96(0.62–13.9	0.06
Vaccination status	Yes	25	1	1	
	No	21	11.9(5.54–25.91)	10.8(4.58–25.48)	0.000
Toilet utilization	Yes	32	1	1	
	No	14	2.24(0.67–7.48)	1.63(0.44–6.1)	0.4
Family size	Maximum five	30	1	1	
	Above five	16	2.01(0.92–4.39)	1.16(0.45–2.99)	0.7
Sex of the child	Female	31	1	1	
	Male	15	0.88(0.40–1.94)	0.59(0.17–2.10)	0.4
Women Empowerment	Low	38	1	1	
	Medium	7	0.24(0.08–0.71)	0.34(0.11–0.88)	0.04
	High	1	0.19(0.02–1.42)	0.33(0.04–2.63)	0.3
Age of the mother	Less than 25	22	1	1	
	25–34	19	1.68(0.39–7.24)	3.72(0.75–18.51)	0.1
	35–44	5	2.43(0.47–12.57)	3.17(0.46–21.45)	0.23

*Others: single, divorced, widowed, and separated

## Discussion

This study was conducted to assess the prevalence of diarrheal disease and associated facotsr among children aged 6–59 months in Oda Bultum District, Ethiopia. Accordingly, the study revealed that the prevalence of diarrhea was 7.4%. This study was lower compared to the study findings from Wolkite town, southern Ethiopia (20.7%) [29], in Sidama Zone, Southern Ethiopia (13.6%) [29], in Horo Guduru Welega Zone, Oromia (24%) [17], and in Rwanda (12.7%) [30], but higher compared to study from Malaysia that found that the prevalence of diarrhea among children was 4.4% [31]. The regional, immunization status and sociocultural variations between our research locations may be the reason for the discrepancy; in our study area, children had high immunization rates, and most of them drank water from pipes.

The study also revealed that children who had not received any vaccination were 10.82 times more likely to have diarrhea compared to their counterparts. This is explicitly related to the fact that vaccination is considered the best preventive intervention of diarrheal diseases. Even having access to clean water, better sanitation, and hygienic practices is not enough to reduce the presence of microorganisms that cause diarrhea, so when a child get vaccinations, it dramatically aids them in fighting against the diarrheal diseases [32]. So, if childhood vaccinations is being administered on time, they help build immunity before children were exposed to fatal illnesses or if they are given at the right age [33–38]. In addition to boosting the immunity of the children to be protective from diarrhea, some vaccines, specifically rotavirus and measles vaccines directly reduces the risk of diarrheal diseases [32, 39–41].

Finally, this study found that children with mothers who had a medium level of empowerment were 66% less likely than children with mothers who had a low level of empowerment to develop diarrhea. This is supported by the fact that the health of mothers and children is closely correlated with mothers’ empowerment, which is in turn correlated with children’s nutritional quality because nutrition conditions are one of the causes of diarrhea among children [42, 43]. Additionally, the relationship between women’s empowerment and children’s health outcomes is related to children’s non-nutrition-related health indicators. Those mothers with good levels of empowerment have the ability to use health services, including vaccinations, which can help the child avoid bacterial, viral, and other related causes of diarrhea [44–47]. It has been shown that when women are empowered, they have the capacity to govern and manage household resources. As a consequence, mothers can afford sanitation materials like water, soap, and other items that are better for preventing and controlling diarrheal disease within the family, including children [48, 49].

## Limitation of this study

Even if this study has some strengths, it cannot be free from limitation. The first limitation of this study could be due to the cross-sectional study nature of lacking cause effect relationship establishment, i.e. unable to establish the temporal relationship between diarrhea and its predictors. Another limitation can be the diarrhea that is considered in this study is any form of diarrheal diseases regardless of their causative agents. The third limitation could be vaccination status of the children was classified as vaccinated or unvaccinated based on any type of vaccines that they received for their age. So, the current study simply showed that whether a child received vaccine recommended for his/her age. Instead, it would be better if the vaccines type were specific and the vaccination status was classified as fully, partially or unvaccinated, but such kind of information was not collected. The fourth limitation could be lack of household/maternal-related factors such as parental vaccines acceptance, personal hygiene, drinking water, and fecal matters handling practices.

## Conclusion

The study found that children who did not receive vaccines were more likely affected by diarrheal disease than those who received the vaccine. As a result, this is directly tied to the fact that vaccination is often recognized as the most effective preventive measure. Even having access to clean water, good sanitation, and hygienic habits are insufficient to diminish the prevalence of germs that cause diarrhea; therefore, immunizations considerably help a child to fight sickness. Moreover, children with mothers who had a medium level of empowerment were less likely to get diarrhea than children with mothers who had a low level of empowerment, according to the study. As a result, this is supported by the fact that maternal and child health is inextricably linked to mothers’ empowerment, which is related to children’s nutritional quality, because problem-related to nutritional status of the children is one of the causes of diarrhea among the children.

## Data Availability

All related data have been presented within the manuscript. The dataset supporting the conclusions of this article is available from the corresponding author upon request.
